# An Interactome-Centered Protein Discovery Approach Reveals Novel Components Involved in Mitosome Function and Homeostasis in *Giardia lamblia*

**DOI:** 10.1371/journal.ppat.1006036

**Published:** 2016-12-07

**Authors:** Samuel Rout, Jon Paulin Zumthor, Elisabeth M. Schraner, Carmen Faso, Adrian B. Hehl

**Affiliations:** 1 Institute of Parasitology, University of Zurich (ZH), Zurich, Switzerland; 2 Institute of Veterinary Anatomy, University of Zurich (ZH), Zurich, Switzerland; Northeastern University, UNITED STATES

## Abstract

Protozoan parasites of the genus *Giardia* are highly prevalent globally, and infect a wide range of vertebrate hosts including humans, with proliferation and pathology restricted to the small intestine. This narrow ecological specialization entailed extensive structural and functional adaptations during host-parasite co-evolution. An example is the streamlined mitosomal proteome with iron-sulphur protein maturation as the only biochemical pathway clearly associated with this organelle. Here, we applied techniques in microscopy and protein biochemistry to investigate the mitosomal membrane proteome in association to mitosome homeostasis. Live cell imaging revealed a highly immobilized array of 30–40 physically distinct mitosome organelles in trophozoites. We provide direct evidence for the single giardial dynamin-related protein as a contributor to mitosomal morphogenesis and homeostasis. To overcome inherent limitations that have hitherto severely hampered the characterization of these unique organelles we applied a novel interaction-based proteome discovery strategy using forward and reverse protein co-immunoprecipitation. This allowed generation of organelle proteome data strictly in a protein-protein interaction context. We built an initial Tom40-centered outer membrane interactome by co-immunoprecipitation experiments, identifying small GTPases, factors with dual mitosome and endoplasmic reticulum (ER) distribution, as well as novel matrix proteins. Through iterative expansion of this protein-protein interaction network, we were able to i) significantly extend this interaction-based mitosomal proteome to include other membrane-associated proteins with possible roles in mitosome morphogenesis and connection to other subcellular compartments, and ii) identify novel matrix proteins which may shed light on mitosome-associated metabolic functions other than Fe-S cluster biogenesis. Functional analysis also revealed conceptual conservation of protein translocation despite the massive divergence and reduction of protein import machinery in *Giardia* mitosomes.

## Author Summary

Organelles with endosymbiotic origin are present in virtually all extant eukaryotes and have undergone considerable remodeling during > 1 billion years of evolution. Highly diverged organelles such as mitosomes or plastids in some parasitic protozoa are the product of extensive secondary reduction. They are sufficiently unique to generate interest as targets for pharmacological intervention, in addition to providing a rich ground for evolutionary cell biologists. The so-called mitochondria-related organelles (MROs) comprise mitosomes and hydrogenosomes, with the former having lost any role in energy metabolism along with the organelle genome. The mitosomes of the intestinal pathogen *Giardia lamblia* are the most highly reduced MROs known and have proven difficult to investigate because of their extreme divergence and their unique biophysical properties. Here, we implemented a novel strategy aimed at systematic analysis of the organelle proteome by iterative expansion of a protein-protein interaction network. We combined serial forward and reverse co-immunoprecipitations with mass spectrometry analysis, data mining, and validation by subcellular localization and/or functional analysis to generate an interactome network centered on a giardial Tom40 homolog. This iterative *ab initio* proteome reconstruction provided protein-protein interaction data in addition to identifying novel organelle proteins and functions. Building on this data we generated information on organelle replication, mitosome morphogenesis and organelle dynamics in living cells.

## Introduction

Since the single endosymbiotic event leading to establishment of mitochondria approximately 2 billion years ago [1,2,3] these organelles have undergone massive changes and have evolved into highly specialized and essential subcellular compartments in all eukaryotes [4,5], with only one possible exception identified so far [6]. These changes comprise a dramatic size reduction, nuclear transfer of organelle genomes, and a renewal of the proteome, which is synthesized almost entirely as precursor proteins on cytosolic ribosomes [7,8,9,10,11,12,13,14] and imported from the cytoplasm [15]. Mitochondria have been remodeled and/or restructured to very different degrees in different species. Mitochondria-related organelles (MROs), *i*.*e*. hydrogenosomes and mitosomes [16,17,18,19,20] in some protists lacking canonical mitochondria represent extreme forms of reduction and/or divergence. The potential of highly diverged organelle-specific pathways as targets for intervention has sparked research into the evolution of MROs in single-celled organisms of all five eukaryotic supergroups [21,22]. Notably, the microaerophilic protozoan pathogens *Entamoeba histolytica* [20] and *Giardia lamblia* [23,24], as well as intracellular parasites such as *Cryptosporidium parvum* [25] and *Encephalitozoon cuniculi* [26] harbor mitosomes. Interestingly, recent investigation of MROs in *Spironucleus salmonicida*, a diplomonad and the closest relative of *G*. *lamblia* belonging to the Excavata super-group, revealed that these organelles are in fact hydrogenosomes [27]. Although it has been demonstrated that *G*. *lamblia* mitosomes do not produce hydrogen, this sheds a completely new light on the evolution of MROs in diplomonads.

Proliferating *G*. *lamblia* trophozoites contain 20–50 double membrane-bounded 100 nm spherical mitosomes [23,24] devoid of an organelle genome [28,29,30,31]. Although not proven experimentally, *G*. *lamblia* mitosomes are likely essential due to a subset of conserved mitochondrial proteins required for iron- sulphur (Fe-S) protein maturation [23,32,33,34,35]. Yeast genetic experiments suggested that Fe-S protein maturation, the only function currently ascribable to *G*. *lamblia* mitosomes, is in fact the minimal essential function of mitochondria [36]. Hence, these organelles have also attracted considerable interest as cell biological models to study extreme reductive evolution of MROs [23,37,38,39,40,41,42]. However, due to massive, albeit selective sequence divergence in *G*. *lamblia*, conventional data mining strategies for identification of mitosome proteins based on homology-based *in silico* searches fall short [26,28,32,43,44,45,46,47]. Moreover, classical, organelle enrichment-based proteome analyses approaches have had only limited success owing to the small size of the organelles and the omnipresence of contaminating endoplasmic reticulum (ER) and cytoskeleton elements in mitosome fractions [33,48,49].

Nevertheless, there is unambiguous experimental evidence for the functional conservation of the mitosomal protein import machinery [20,23,24,49]. The small subset of structurally conserved mitosome proteins such as *G*. *lamblia* IscU, ferredoxin, Cpn60, IscS and mtHsp70 are imported by transit peptide-dependent and -independent mechanisms [23]. However, the predicted components of the TOM/TIM import apparatus are diverged beyond recognition by state-of-the-art homology search tools. Indeed, the protein repertoire of the mitosomal outer membrane and its networks are scarcely characterized: only one subunit of the translocon in the outer mitochondrial (TOM) complex, a highly diverged Tom40 homologue (*Gl*Tom40), and [50] more recently a giardial Tim44 homologue [49], have been identified. Furthermore, there is no information on how mitosome homeostasis is achieved in terms of organelle size and number.

To address questions concerning protein networks at mitosomal membranes in association with mitosome homeostasis and to account for the extreme sequence divergence in *G*. *lamblia*, we implemented novel experimental approaches. We were successful to tag two outer membrane organelle proteins with GFP to show that these small organelles are immobilized, distinctive entities with no appreciable inter-organelle exchange or network character. Using a giardial TOM40 homolog as a starting bait we generated information on protein-protein interactions at the outer membrane as well as expanding the organelle proteome by identifying novel components. By using interactome targets validated by subcellular localization as baits for subsequent reverse co-IP rounds, we were able to extend this initial interactome beyond the outer membrane, including dually localized endoplasmic reticulum (ER) and mitosome proteins, as well as identifying previously described and novel imported organelle proteins. In addition to identification of two components with a role in mitosome morphogenesis and homeostasis the combined data revealed a core organelle membrane interactome composed of only 3 tightly-associated proteins. Furthermore, we tested constraints for import of nuclear-encoded mitosome proteins and could show conservation of this mechanism even in the highly diverged and reduced *Giardia* mitosome.

## Materials and Methods

### *Giardia* cell culture, induction of encystation, pulse-empty chase set-up and transfection

*G*. *lamblia* WB (line C6; ATCC catalog number 50803) trophozoites were grown and harvested using standard protocols [51]. Encystation was induced with the two-step method as described previously [40,52]. Transgenic parasites were generated according to established protocols by electroporation of linearized pPacV-Integ-based plasmid vectors prepared from *E*. *coli* as described in [42]. After selection for puromycin resistance, transgenic *G*. *lamblia* cell lines were cultured without puromycin.

### Construction of expression vectors

All sequences of oligonucleotide primers for PCR used in this work are listed in S1 Table.

For cloning of C-terminally hemagglutinin (HA)-tagged proteins in *Giardia*, a vector PAC-CHA was designed on the basis of the previously described vector pPacV-Integ [42], where additional restriction sites were inserted [53].

A cyst wall protein 1 promoter (pCWP1)-driven *G*. *lamblia* ferredoxin (fd)-human dihydrofolate reductase (DHFR) chimeric gene was generated by fusing two genes by overlapping PCR: i) an intron-less fd mitosomal targeting signal (MTS) (MTSfdΔ_int_) open reading frame (ORF) was generated using primer pair 33 (S1 Table) with *G*. *lamblia* cDNA as template, ii) a DHFR_HA minigene was generated using primer pair 34 (S1 Table) with a cloned human DHFR cDNA as template. The fused product was digested with *Spe*I and *Pac*I and inserted in a PAC vector to yield construct pCWP1_MTSfdΔ_int_-DHFR_HA.

A pCwp1_ MTSfdΔ_int_-DHFR_Neomycin resistance construct (without HA tag) was generated for protein import block assays. Primer pair 35 (S1 Table) was used on pCwp1_ MTSfdΔ_int_-DHFR_HA as a template. The amplified product was digested with *Nsi*I and *Pac*I and ligated into a vector containing a neomycin resistance cassette [51].

### Co-immunoprecipitation with limited cross-linking

*G*. *lamblia* WBC6 and transgenic trophozoites expressing C-terminally HA tagged bait proteins were harvested and subjected to immunofluorescence assay to confirm correct subcellular distribution of bait proteins. Parasites were collected by centrifugation (900 x *g*, 10 minutes, 4°C), washed in 50 ml of cold phosphate buffer saline solution (PBS) and adjusted to 2 x10^7^ cells^.^ml^-1^ in PBS (VWR Prolabo). The appropriate formaldehyde concentration for cross-linking (2.25%) was determined by a titration assay (S2 Fig). For the co-immunoprecipitation (co-IP) assays, 10^9^ parasites were resuspended in 10 ml 2.25% formaldehyde (in PBS) supplemented with 1 mM phenylmethylsulfonyl fluoride (PMSF; SIGMA, Cat. No. P7626) and incubated for 30 minutes at room temperature (RT). Cells were pelleted, washed once with 10 ml PBS, and quenched in 10 ml 100 mM glycine in PBS for 15 minutes at RT. The collected cells were then resuspended in 5 ml RIPA lysis buffer (50 mM Tris pH 7.4, 150 mM NaCl, 1% IGEPAL, 0.5% sodium deoxycholate, 0.1% SDS, 10 mM EDTA) supplemented with 2 mM PMSF and 1 x Protease Inhibitor cocktail (PIC, Cat. No. 539131, Calbiochem USA) and sonicated twice using a Branson Sonifier with microtip (Branson Sonifier 250, Branson Ultrasonics Corporation) with the following settings: 60 pulses, 2 output control, 30% duty cycle and 60 pulses, 4 output control, 40% duty cycle. The sonicate was incubated on a rotating wheel for 1 h at 4°C, aliquoted into 1.5 ml tubes and centrifuged (14,000 x *g*, 10 minutes, 4°C). The soluble protein fraction was mixed with an equal volume detergent-free RIPA lysis buffer supplemented with 2% TritonX (TX)-100 (Fluka Chemicals) and 40 μl anti-HA agarose bead slurry (Pierce, product # 26181). After binding of tagged proteins to the beads at 4°C for 2 h on a rotating wheel, beads were pulse-centrifuged and washed 4 times with 3 ml Tris-Buffered Saline (TBS) supplemented with 0.1% TX-100 at 4°C. After a final wash with 3 ml PBS the loaded beads were resuspended in 350 μl PBS, transferred to a spin column (Pierce spin column screw cap, product # 69705, Thermo Scientific) and centrifuged for 10 s at 4°C. Elution was performed by resuspending beads in 30 μl of PBS. Dithiothreitol (DTT; 100mM; Thermo Scientific, Cat. # RO861) was added and samples were boiled for 5 min followed by centrifugation (14,000 x *g*, 10 minutes, RT).

### Protein analysis and sample preparation for mass spectrometry-based protein identification

SDS-PAGE and immunoblotting analysis of input, flow-through, and eluate fractions was performed on 4%-12% polyacrylamide gels under reducing conditions, (molecular weight marker Cat. No. 26616, Thermo Scientific, Lithuania). Transfer to nitrocellulose membranes and antibody probing were done as described previously [54], using anti-HA (dilution 1:500; Roche) followed by anti-rat antibodies coupled to horseradish peroxidase (dilution 1:5000; Southern Biotech). Gels for mass spectrometry (MS) analysis were stained using Instant blue (Expedeon, Prod. # ISB1L) and de-stained with sterile water.

### Mass Spectrometry, protein identification and data storage

Stained gel lanes were cut into 8 equal sections. Each section was further diced into smaller pieces and washed twice with 100 μl of 100 mM ammonium bicarbonate/ 50% acetonitrile for 15 min at 50°C. The sections were dehydrated with 50 μl of acetonitrile. The gel pieces were rehydrated with 20 μl trypsin solution (5 ng/μl in 10 mM Tris-HCl/ 2 mM CaCl_2_ at pH 8.2) and 40 μl buffer (10 mM Tris-HCl/ 2 mM CaCl_2_ at pH 8.2). Microwave-assisted digestion was performed for 30 minutes at 60°C with the microwave power set to 5 W (CEM Discover, CEM corp., USA). Supernatants were collected in fresh tubes and the gel pieces were extracted with 150 μl of 0.1% trifluoroacetic acid/ 50% acetonitrile. Supernatants were combined, dried, and the samples were dissolved in 20 μl 0.1% formic acid before being transferred to the autosampler vials for liquid chromatography-tandem MS (injection volume 7 to 9 μl). Samples were measured on a Q-exactive mass spectrometer (Thermo Scientific) equipped with a nanoAcquity UPLC (Waters Corporation). Peptides were trapped on a Symmetry C18, 5 μm, 180 μm x 20 mm column (Waters Corporation) and separated on a BEH300 C18, 1.7 μm, 75 μm x 150 mm column (Waters Corporation) using a gradient formed between solvent A (0.1% formic acid in water) and solvent B (0.1% formic acid in acetonitrile). The gradient started at 1% solvent B and the concentration of solvent B was increased to 40% within 60 minutes. Following peptide data acquisition, database searches were performed using the MASCOT search program against the *G*. *lamblia* database (http://giardiadb.org/giardiadb/) with a concatenated decoy database supplemented with commonly observed contaminants and the Swissprot database to increase database size. The identified hits were then loaded onto the Scaffold Viewer version 4 (Proteome Software, Portland, US) and filtered based on high stringency parameters, i.e. 95% for peptide probability, a protein probability of 95%, and a minimum of 2 unique peptides per protein. Where indicated in the text, slightly relaxed filtering parameters were applied. Proteins identified in both bait-specific and control datasets were considered of interest if they were at least 5-fold enriched in the bait-specific datasets (in terms of spectral counts) based on high stringency parameters. Access to raw MS data is provided through the ProteomeXchange Consortium on the PRIDE platform [55].

### *In silico* co-immunoprecipitation dataset analysis

Analysis of primary structure and domain architecture of putative mitosomal hypothetical proteins was performed using the following tools and databases: MITOPROT (https://ihg.gsf.de/ihg/mitoprot.html) and PSORTII (http://psort.hgc.jp/form2.html) for subcellular localization prediction, TMHMM (http://www.cbs.dtu.dk/services/TMHMM/) for transmembrane helix prediction, SMART (http://smart.embl-heidelberg.de/) for prediction of patterns and functional domains, pBLAST for protein homology detection (http://blast.ncbi.nlm.nih.gov/Blast.cgi?PAGE=Proteins), HHPred (http://toolkit.tuebingen.mpg.de/hhpred) for protein homology detection based on Hidden Markov Model (HMM-HMM) comparison, and the Giardia Genome Database (http://giardiadb.org/giardiadb/) to extract other/organism-specific information, e.g. expression levels of the protein, predicted molecular size and nucleotide/protein sequence. For functional domains predicted by SMART we used an e-value of 10e^-5^ as cutoff, and for protein homologies predicted by pBLAST we accepted alignment scores above 80. However, since G. *lamblia* homologs for eukaryotic proteins are highly diverged, we also considered functional domain predictions associated to a lower e-value. Alignment scores between 50 and 80 were accepted only when pBLAST predictions were consistent with HHPred output.

### Immunofluorescence analysis (IFA) and microscopy

Preparation of chemically fixed cells for immunofluorescence and analysis of subcellular distribution of reporter proteins by wide-field and confocal microscopy were done as described previously [42,54]. Nuclear labelling was performed with 4',6-diamidino-2-phenylindole (DAPI). The HA epitope tag was detected with a monoclonal anti-HA antibody coupled to FITC (dilution 1:50; Roche) whereas *Gl*IscU was detected with a self-made antibody (dilution 1:300) followed by an anti-mouse antibody coupled to Alexafluor 594 (dilution 1:300; Molecular Probes). To avoid any possibility for cross reaction, co-labelling experiments for IFA were performed by incubating first with the anti-*Gl*IscU antibody, followed by the AF594-conjugated anti-mouse secondary antibody, and direct detection of the HA epitope tag with a FITC-conjugated rat anti-HA monoclonal antibody as a final step.

### Live-cell microscopy and fluorescence recovery after photobleaching (FRAP)

Transgenic *G*. *lamblia* trophozoites expressing GFP-*Gl*Tom40 or *Gl*29147-GFP were harvested and prepared for imaging in PBS supplemented with 5 mM glucose (Cat. No. 49139, Fluka), 5 mM L-cysteine (Cat. No. C6852, Sigma) and 0.1 mM ascorbic acid (Cat. No. 95209, Fluka) at pH 7.1. FRAP and time-lapse series were performed as described previously [54,56].

### Sample preparation for transmission electron microscopy

Transgenic trophozoites ectopically expressing wild type *G*. *lamblia* dynamin related protein (*Gl*DRP) (ORF *Gl50803_14373*) or the constitutively active (GTP-locked) *Gl*DRP-K43E variant under the control of the CWP1 promoter [56] were harvested 3 h post induction and analyzed by transmission electron microscopy (TEM) as described previously [56].

### Sub-cellular fractionation analysis

For sub-cellular fraction experiments, 4^.^10^6^ *Gl*DRP-HA and *Gl*DRP-K43E-HA- expressing transgenic cells were lysed by freeze-thawing and supernatant (soluble fraction) and pellet (membrane fraction) were prepared by centrifugation at 14’000 x *g* for 10 minutes at 4°C. The HA-tagged proteins were detected by SDS-PAGE and Western blot using a rat anti-HA mAb (clone 3F10, Roche) as described previously [54].

### DHFR-MTX protein import block assay

The MTSfdΔ_int_-DHFR fusion (see also above under “Constructs”) was expressed under the control of the inducible CWP1 promoter in a background transgenic line constitutively expressing HA- tagged 17030 (cell line Cwp1_MTSfdΔ_int_-DHFR/*Gl*17030HA). DHFR expression was induced using the 2-step method [40] for 4 h and “chased” for 24 h by placing the cells again in standard growth medium in the presence or absence of 1 μM methotrexate (MTX). Total cell lysates were separated by SDS-PAGE and Western blot to detect processed and unprocessed forms of the *Gl*17030HA reporter. Subcellular distribution was analyzed by immunofluorescence assay (IFA) using wide field microscopy.

## Results

### *G*. *lamblia* mitosomes do not form dynamic networks and are associated to the single dynamin-related protein *Gl*DRP

**Fig 1:**
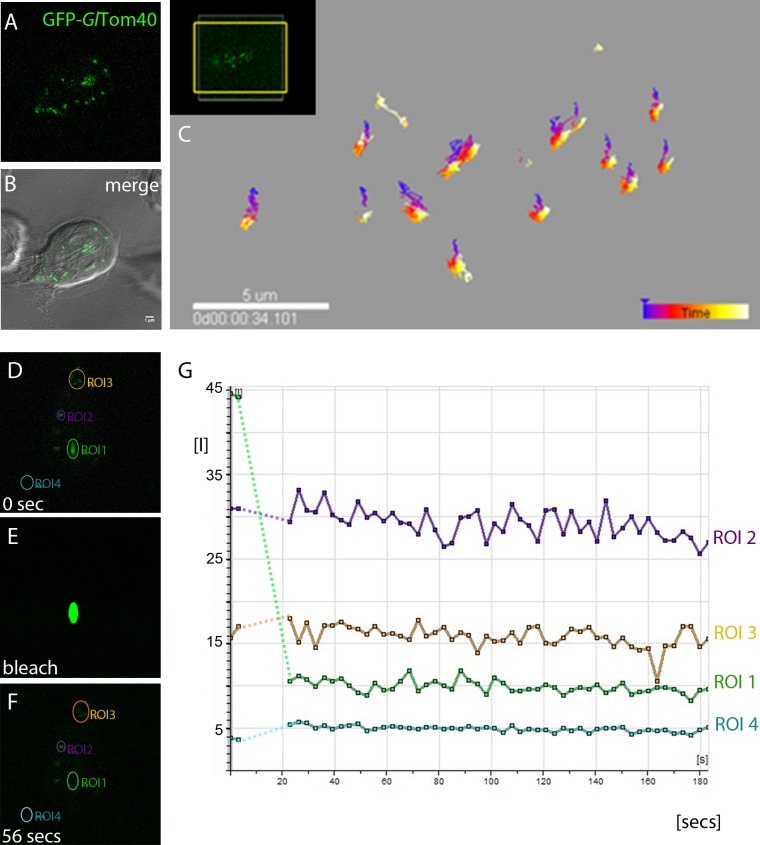
G. lamblia mitosomes are immobilized and do not form dynamic networks. (A-B) Detection of typical organelle distribution of GFP-tagged *Gl*Tom40 (green) in time-lapse microscopy. (B) Overlay showing the CMC between the two nuclei and PMs which show a canonical dispersed localization throughout the cell. (C) Tracking of organelles during a period of 30 min shows no significant movement of mitosomes in the cytosol. (D-G) FRAP experiments performed on cells conditionally expressing GFP-tagged *Gl*Tom40 suggest that outer membrane proteins are not able to move amongst/in between organelles. (E) Photobleaching of a single mitosome (region of interest 1 (ROI 1)) in a living cell is shown. (F-G) Fluorescence in a bleached organelle (green line in the graph) does not recover even after several minutes (>20 min). Purple and brown lines in the graph represent fluorescence in unbleached areas (ROIs 2 and 3). (G) Fluorescence micrographs from the image series at the start (0 sec) of the experiment, during bleaching, and at the beginning of the recovery phase (20 sec). Arbitrary units of fluorescence are indicated [I]. Broken lines connect pre- and post-bleaching values in the graph. Scale bar: 1 μM.

Mitochondria in higher eukaryotes are highly dynamic organelle networks that move in the cell via microtubules and microfilaments and undergo constant fission and fusion to meet the energy requirements of the cell [57,58]. IFA and TEM analyses suggest that *G*. *lamblia* mitosomes are very small spherical organelles with no evidence of network formation. In addition, the mitosome population in each cell can be divided into peripheral mitosomes (PM) distributed randomly in the cytoplasm and what has been dubbed the central mitosome complex (CMC) [23]. The latter consists of a grape-like cluster of individual organelles of the size and shape of peripheral mitosomes that is closely and permanently associated to the basal body complex between the two nuclei [23]. Interestingly, these organelles remain spatially distinct despite their close proximity. The motility of this central cluster is highly constrained and restricted to ordered segregation with the duplicated basal body complex during cell division [23]. Because green fluorescent protein (GFP) imported into the mitosome matrix is not fluorescent [23], GFP-tagging of mitosomes has not been possible until now. Martinkova et al. [59] have shown that mitosomes in trophozoites can be labeled for live cell microscopy using HaloTag markers [60]. However, no quantitative information on the spatial dynamics of peripheral mitosomes in the cytoplasm was presented in this report. We investigated organelle dynamics in living cells by performing time lapse microscopy of cells expressing GFP-tagged mitosome reporters for the outer membrane. Conditional expression of N-terminally GFP-tagged *Gl*Tom40 with 3 h of induction followed by “chasing” newly-synthesized GFP-Tom40 into mitosomes over 2–3 h in normal conditions was found suitable for labeling organelles in living cells (Fig 1A and 1B). Tracking of individual organelles over a period of >30 min showed no significant cytoplasmic movement or changes in number or morphology (Fig 1C), suggesting that organelles neither move randomly nor are they transported directionally in the cytoplasm along cytoskeleton structures. To test whether mitosome outer membrane proteins are exchanged between organelles we performed FRAP experiments on cells conditionally expressing *Gl*Tom40-GFP. Since *Gl*Tom40-GFP is membrane-anchored, FRAP addresses the question whether mitosomes are isolated organelles and whether they form membrane continuities which would allow exchange of outer membrane proteins. No recovery of fluorescence in bleached CMC or PM organelles was detected (Fig 1D–1G) suggesting that peripheral and CMC organelle membranes remain distinct.

**Fig 2:**
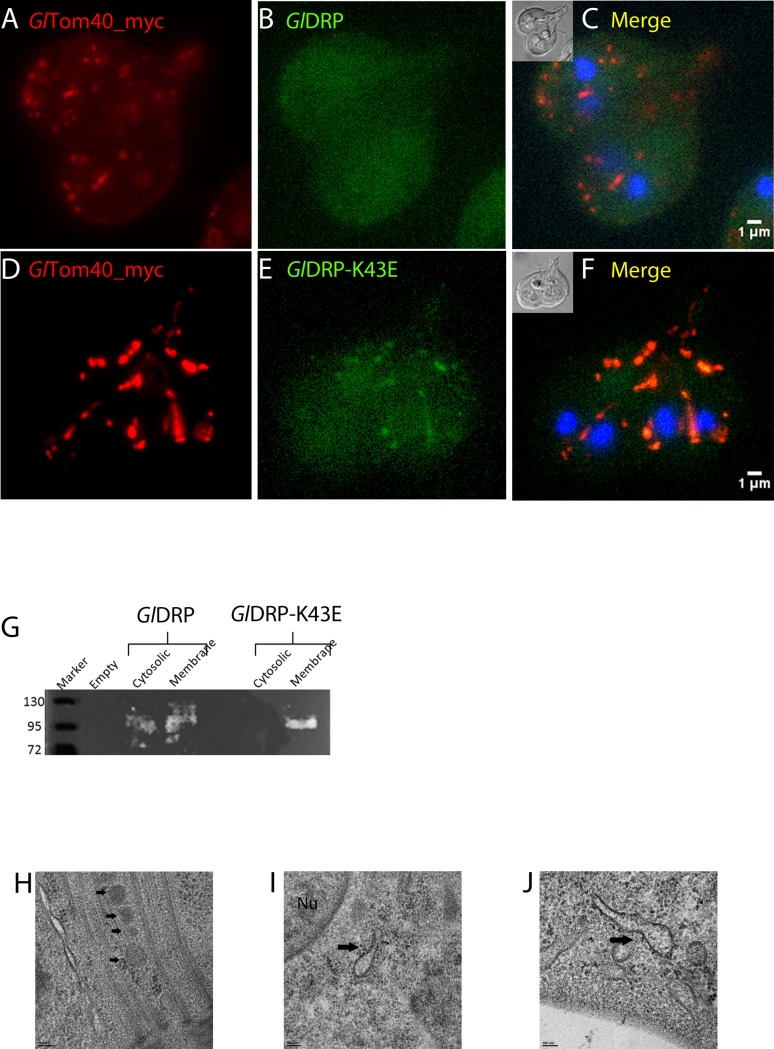
Conditional expression of GlDRP-K43E elicits a mitosome morphogenesis phenotype. (A-C) Subcellular localization of a C-myc tagged *Gl*Tom40 (red) by IFA in cells induced to express a wild type *Gl*DRP (green) or GTP-locked variant *Gl*DRP-K43E (D-F). Note the altered size and distribution of organelles labeled with Tom40-myc in *Gl*DRP-K43E expressing lines. Nuclear DNA is stained with DAPI (blue). Insets: DIC images. Scale bar: 1 μM (G) -Cell fractionation experiments confirm fixed membrane localization of *Gl*DRP-K43E. (H) TEM: normal morphology of mitosomes (black arrows) in the CMC in cells expressing wild type *Gl*DRP whilst cells expressing *Gl*DRP-K43E show enlarged dumbbell-shaped mitosomes (black arrows in I, J) indicative of defective organelle division. Nu: nucleus. Scale bars: 100 nm.

Despite intensive research in the field of MROs, little is known regarding factors required for their division. Dynamin-related proteins (DRPs) are implicated in mitochondrial and hydrogenosomal division in higher eukaryotes and in protozoa such as *Trypanosoma brucei* [61,62] and *Trichomonas vaginalis* [63]. *G*. *lamblia* harbors a single DRP (ORF *Gl50803_14373*) [56] with a previously documented role in trafficking of cyst wall material, and endocytic and exocytic organelle homeostasis [56]. To test for a hitherto unrecognized role of *Gl*DRP in determining mitosome morphology and/number, we used a dual cassette expression vector [54] to express constitutive C-terminally myc-tagged *Gl*Tom40 as a reporter for mitosomes and inducible C-terminally HA-tagged wild-type (*Gl*DRP-HA) or GTP-locked (*Gl*DRP-K43E-HA) variants in trophozoites. In trophozoites expressing *Gl*DRP-HA (Fig 2A–2C), IFA analyses demonstrated the typical random cytoplasmic distribution of PMs i.e. “dispersed” [23]. However, cells expressing the GTP-locked variant *Gl*DRP-K43E-HA (Fig 2D–2F) presented a “clustered” mitosome phenotype, indicative of enlarged organelles. Consistent with this phenotype and in line with previous reports [56], the subcellular distribution of HA-tagged *Gl*DRP remained mostly cytosolic (Fig 2B). Conversely, *Gl*DRP-K43E-HA showed a punctate distribution (Fig 2E) and significant signal overlap with *Gl*Tom40-myc (Fig 2F), suggesting selective accumulation of *Gl*DRP-K43E-HA on mitosome membranes. We tested whether this marked association of ectopically expressed *Gl*DRP-K43E with organelle membranes compared to the wild type DRP variant in IFA could be corroborated in cell fractionation experiments. Separation by SDS-PAGE and immunoblot analysis revealed that *Gl*DRP-HA was almost equally distributed between the “cytosolic” and “membrane” fraction, whereas the mutated variant *Gl*DRP-K43E-HA was detected only in the “membrane” fraction (Fig 2G). These data were consistent with the microscopical analysis in Fig 2E and suggest increased association of *Gl*DRP-K43E-HA with organelle membranes compared to wild-type *Gl*DRP-HA. To characterize the nature of the *Gl*DRP-K43E-HA-dependent phenotype in more detail, we performed transmission electron microscopy of induced transgenic cells. Cells expressing the *Gl*DRP-K43E-HA variant frequently presented elongated and tubular mitosome structures (Fig 2I and 2J) compared to cells expressing wild type *Gl*DRP-HA (Fig 2H).

Taken together, these data show how mitosomes are immobilized in the cell and present no measurable outer membrane exchange in the conditions tested. Their morphogenesis is perturbed following conditional ectopic expression of a dominant-negative GTP-locked *Gl*DRP variant, suggesting a previously unappreciated role for this GTPase in the maintenance of mitosome integrity and organelle morphogenesis in *G*. *lamblia*.

### Co-IP with the *G*. *lamblia* Tom40 homolog identifies novel interacting proteins in the mitosome outer membrane

**Fig 3:**
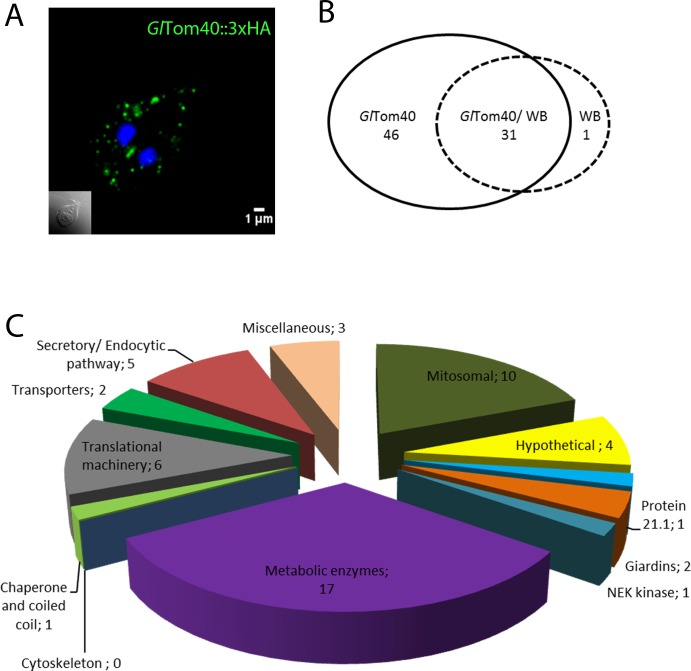
Co-IP with HA_tagged GlTom40 yields numerous candidate interacting proteins. (A) Immunofluorescence microscopy: C-terminally HA-tagged *Gl*Tom40 (*Gl*Tom40-HA) is an exclusive marker for mitosomes (green). Nuclear DNA is stained with DAPI (blue). Inset: DIC image. (B) Venn diagram indicating 46 *Gl*Tom40 specific hits. (C) Parsing of 46 *Gl*Tom40-specific and 6 enriched proteins in metabolic categories based on available annotations in www.giardiaDB.org.

The aberrant mitosome morphology after conditional expression of *Gl*DRP-K43E points towards mitosome-associated machinery at the organelle’s surface involved in organelle homeostasis. Despite efforts aimed at defining the protein content of mitosomes in *Giardia* [33,49,50], the composition of this organelle’s outer and inner membrane proteome remains sparsely characterized, with the exception of a highly diverged putative Tom40 homologue (*Gl*Tom40; ORF *Gl50803_17161*) and a structurally-conserved Tim44 [49,50]. To generate a robust mitosome outer membrane proteome we focused on *Gl*Tom40 as a point of origin and developed a tailored co-IP protocol with an HA-tagged variant as “bait”. A transgenic line *Gl*Tom40-HA constitutively expressing the epitope-tagged bait protein was generated; exclusive mitosome localization of the bait protein in transgenic cells was confirmed by IFA in co-labelling experiments with a newly-made anti-*Gl*IscU antibody (Fig 3A and S1A Fig). To ensure solubilization of mitosomal membranes while avoiding disruption of Tom40-associated protein complexes, we used carefully titrated, formaldehyde-based cross-linking [64] to stabilize predicted protein-protein interactions in co-IP experiments during extraction with the option to reverse covalent bonds (S2 Fig; see also in Materials and Methods). Following MS analysis and data filtration using a control dataset obtained from non-transgenic cells (ctrl.co-IP) we identified a total of 52 proteins, 46 exclusive and 6 enriched in the *Gl*Tom40 co-IP dataset (Fig 3B). This protein set was parsed and subdivided into different metabolic and/or functional categories (Fig 3C). In the mitosomal protein category few detected four previously identified mitosome proteins namely: mitochondrial HSP70 (ORF *Gl50803_14581*), oxidoreductase 1 (*Gl*OR1; ORF *Gl50803_91252*), proteins Gl50803_9296 and Gl50803_14939, recently named MOMP35 [33,49]. We extracted additional information from the *Gl*Tom40 co-IP data by relaxing stringency parameters to (95_1_95), obtaining a total of 150 proteins (FDR 3.4%). Of these, 109 hits were exclusive to the expanded *Gl*Tom40 co-IP dataset which contained 3 additional annotated mitosome proteins namely, chaperonin 60 (Cpn60; ORF *Gl50803_103891*), *Gl*Qb-SNARE 3 (putative Sec20, ORF *Gl50803_5161*) and *Gl*IscU (NifU-like protein; ORF *Gl50803_15196*).

### Imaging-based validation of the *Gl*Tom40 co-IP dataset

**Fig 4:**
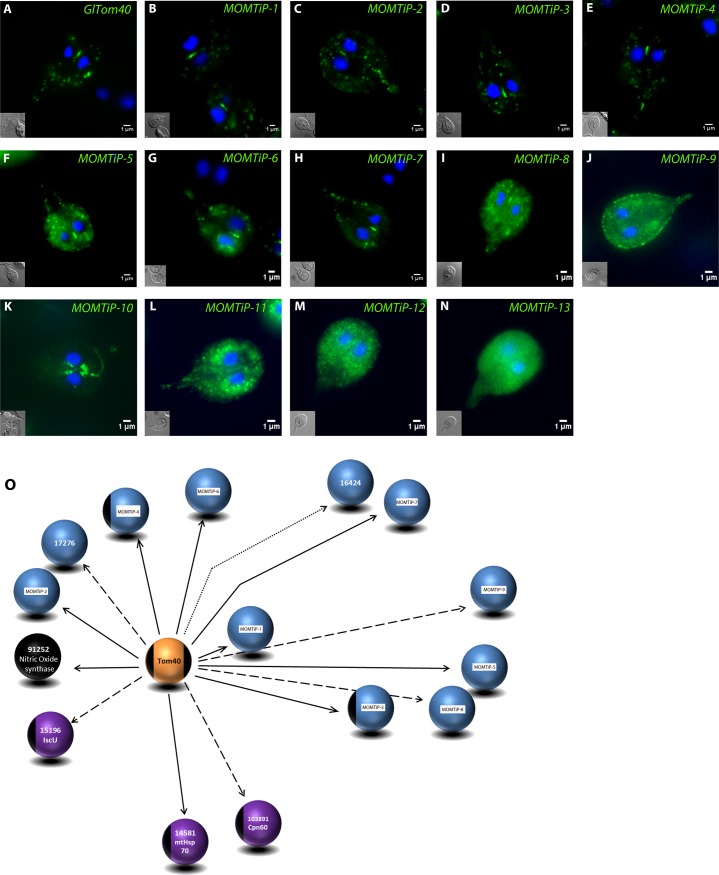
Subcellular localization of co-precipitated GlTom40 interaction partners. (A-N) Immunofluorescence microscopy: subcellular localization of C-terminally HA-tagged *Gl*Tom40 and 13 putative interaction partners (green) falls into 3 categories: Typical mitosome localization (A-E); dual localization to mitosomes and ER (F-I); no or ambiguous mitosome localization (J-N). Nuclear DNA is stained with DAPI (blue). Insets: DIC image. Scale bars: 1μm. (O) Partially validated *Gl*Tom40 interactome showing the bait protein (orange sphere), matrix proteins (purple), previously identified and mitosome-localized proteins (black), and mitosome-localized hypothetical proteins (blue). The stringency parameters used for detection (high, medium, and relaxed) are represented by bold, dashed, and dotted arrows, respectively.

Limited chemical cross-linking in co-IP assays expands the range of discovery beyond primary interactions with the bait. We therefore performed an initial validation of the predicted *Gl*Tom40 interacting proteins in this dataset by subcellular localization of ectopically expressed, epitope-tagged candidates to mitosomes. We selected 13 of the 109 candidate Mitosomal Outer Membrane Tom40 interacting proteins (MOMTiP; Table 1) based on their spectral counts with high stringency parameters and/or protein domains identified with HHPred (S2 Table) and engineered endogenous promoter-driven, C-terminally HA-tagged variants for all. IFA analysis of corresponding transgenic lines showed mitosomal localization for 8 candidates (Fig 4A–4H), of which 4 proteins of unknown function (MOMTiP-5 to 8*)* presented dual localization (mitosome and ER) (Fig 4F–4I). The five remaining proteins of this set of 13 candidates (MOMTiP- 9–13; Fig 4J–4N) showed dispersed patterns of subcellular distribution and were not considered mitosome proteins. Fig 4O shows a consolidated depiction of a first *Gl*Tom40-centered mitosomal outer membrane interactome, which includes the 8 proteins localized to mitosomes described above, as well as 4 previously identified matrix proteins and 3 newly validated hypothetical proteins comprised in the list of *Gl*Tom40 interacting proteins. Taken together, the imaging data are in agreement with the protein-protein interaction data, and support limited chemical crosslinking as a suitable method to stabilize protein complexes during co-IP.

**Table 1:** Selected MOMTiP proteins for validation by light microscopy.

ORF number	Assigned name	Peptide count in *Gl*Tom40-specific co-IP dataset (none detected in WB)
*Gl50803_29147*	MOMTiP-1	30
*Gl50803_10971*	MOMTiP-2	63
*Gl50803_14939*	MOMTiP-3 (GiMOMP35) [49]	21
*Gl50803_9296*	MOMTiP-4	11
*Gl50803_21943*	MOMTiP-5	13
*Gl50803_22587*	MOMTiP-6	8
*Gl50803_5785*	MOMTiP-7 (Qb-SNARE 4) [65]	11
*Gl50803_9503*	MOMTiP-8	1
*Gl50803_7188*	MOMTiP-9	1
*Gl50803_114546*	MOMTiP-10	1
*Gl50803_113892*	MOMTiP11	43
*Gl50803_9719*	MOMTiP-12	47
*Gl50803_10822*	MOMTiP-13	3

### Iterative reverse co-IP experiments expand the mitosomal protein interactome network beyond the outer membrane

IFA analysis of MOMTiP-1 to 13 indicated that the majority of these proteins are associated to mitosomes, thereby providing preliminary validation of the selected 13 candidates of the primary *Gl*Tom40-specific co-IP dataset. To further test the robustness of this primary interactome and expanding it beyond the mitosomal membrane, we performed a first reverse co-IP experiment using MOMTiP-1 (ORF *Gl50803_29147*) as bait. MOMTiP-1 was chosen because it presented the largest spectral count with high stringency parameters in the *Gl*Tom40 dataset and localized unequivocally to mitosomes (S1B Fig).

**Fig 5:**
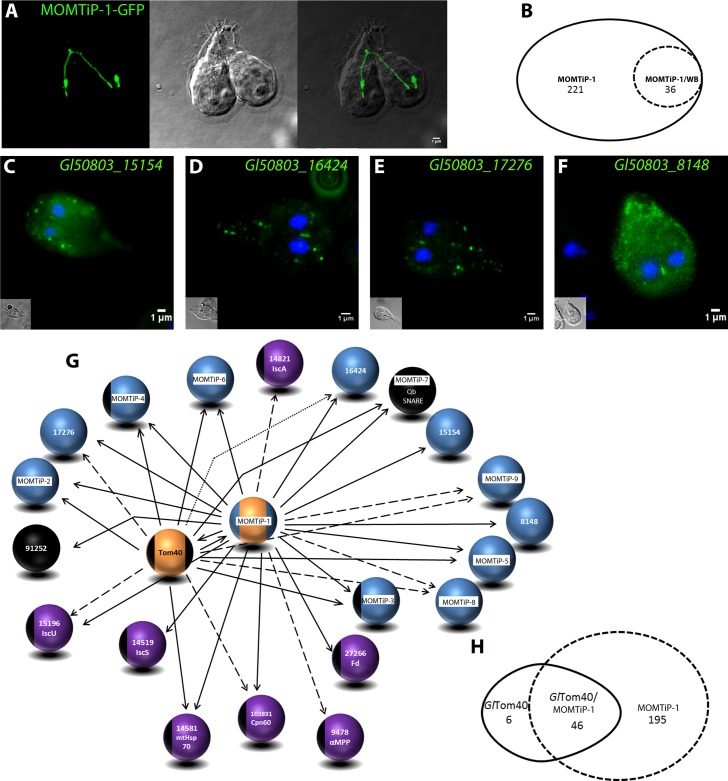
Expansion and validation of the GlTom40 interactome by reverse co-IP with MOMTiP-1. (A) The “string” mitosome phenotype observed upon constitutive expression of GFP-tagged MOMTiP-1 (MOMTiP-1-GFP). Left panel: MOMTiP-1-GFP localized exclusively to mitosomes and in some cases virtually all of the peripheral organelles have been replaced by a single long tubular mitosome spanning both daughter cells length-wise. Middle panel: DIC image. Right panel: Overlay of the two channels. (B) Venn diagram showing MOMTiP-1-specific proteins identified after filtering the dataset. (C-F) Subcellular localization of selected C-terminally HA-tagged novel hypothetical proteins by IFA (green). Nuclear DNA is stained with DAPI (blue). Insets: DIC images. (G) Preliminary interactome of *Gl*Tom40 and MOMTiP-1 showing validated hits. Bait proteins (orange spheres), matrix proteins (purple), previously identified and localized proteins (black), and localized hypothetical proteins (blue). The stringency parameters used for detection (high, medium, and relaxed) are represented by bold, dashed, and dotted arrows, respectively. (H) Venn diagram showing the intersection of *Gl*Tom40 and MOMTiP-1datasets.

**MOMTiP-1** is a *Giardia*-specific mitosome-localized protein of unknown function. *In silico* analysis using TMHMM robustly detected a 22 amino acid-long transmembrane helix in the N-terminal part of the protein followed by a large C-terminal domain predicted to be exposed to the cytosol on the mitosomal surface. To track this protein *in vivo*, we engineered MOMTiP-1 constructs for live cell imaging using GFP reporters. We have shown previously that GFP only fluoresces if exposed to the cytoplasm and never after import into mitosomes [23]. Therefore, the brightly fluorescing and mitosome-localized MOMTiP-1-GFP fusion supports the predicted topology for MOMTiP-1 as a type 1 transmembrane protein with respect to the outer mitosomal membrane. Surprisingly, many cells expressing MOMTiP-1-GFP showed a mitosome morphology dubbed “string” phenotype suggestive of extensive elongation of organelles to large tubules (Fig 5A; left). In many cases, virtually all PMs had been replaced by a single long organelle with a diameter that corresponded to that of an individual mitosome. Although the “string” mitosome phenotype was compatible with survival of the parasites, many trophozoites appeared to be delayed or even arrested in cytokinesis and had a typical heart-shaped appearance (Fig 5A; middle) previously observed in cells which are unable to complete cytokinesis [66]. Because the tubular organelles ran through the non-divided part connecting both daughter cells, we postulated that inability to divide mitosomes impairs completion of cytokinesis.

Co-IP with an HA-tagged variant of MOMTiP-1 yielded a large dataset of 221 exclusive hits (Fig 5B) which included *Gl*Tom40 detected at high stringency parameters, thereby confirming the strong interaction between *Gl*Tom40 and MOMTiP-1. The 221 MOMTiP-1 co-IP specific hits and an additional 20 enriched candidates were parsed according to different metabolic and/or functional categories (Fig 5B). In addition to *Gl*Tom40, the dataset contained several known mitosomal proteins, including matrix proteins HSP70 and *Gi*OR1, cysteine desulfurase (IscS; *Gl50803_14519*), Cpn60, [2Fe-2S] ferredoxin (*Gl50803_27266*) and NifU-like protein, along with all 8 hypothetical proteins previously identified in the *Gl*Tom40 co-IP dataset and 4 additional non-annotated candidate mitosome proteins (Fig 5C–5F). Similarly to MOMTiP-1, one of these (*Gl50803_17276*) is also predicted to carry a TMD close to its N-terminus. Furthermore, this dataset contained two axoneme-associated GASP-180 proteins (*Gl50803_137716* and *Gl50803_16745*) [67] detected with high stringency parameters, in line with association of the CMC to basal bodies.

Taken together, a first reverse co-IP analysis using the single-pass transmembrane MOMTiP-1 provided robust validation of the experimental approach used to identify mitosome membrane proteins, and has expanded the predicted mitosomal membrane and import machinery interactome to 22 proteins (Fig 5G).

Reverse co-IP using MOMTiP-1 as bait demonstrated that this protein and *Gl*Tom40 are strong interaction partners. We analyzed the intersection of their respective datasets to identify common candidate interaction partners and identified 27 proteins with high reliability (Fig 5H), 10 of which localized to mitosomes (Fig 4). Given MOMTiP-1’s predicted topology, strong interaction with *Gl*Tom40 and the interactome overlap, we postulated that MOMTiP-1 and *Gl*Tom40 exist in a core complex mostly likely involved in protein translocation across the outer mitosomal membrane. To characterize other components of this core interactome of the outer mitosomal membrane and to move beyond individual complexes to explore the boundaries of the growing protein interactome network (Fig 5G), we performed a series of additional reverse co-IP experiments using HA-tagged Qb-SNARE 4 (MOMTiP-7), *Gl*IscS, protein *Gl50803_9296* (MOMTiP-4) and protein *Gl50803_14939* (MOMTiP-3) as baits. MOMTiP-7 (Qb-SNARE 4), MOMTiP-4 and MOMTiP-3 were chosen because they were identified either exclusively or in both the *Gl*Tom40- and MOMTiP-1 co-IP datasets, suggesting they may reside in the mitosomal outer membrane and could thus serve as tools for a lateral and outward expansion of this compartment’s interactome. On the other hand, *GI*IscS was chosen to extend the mitosomal proteome inwards towards the organellar matrix. Correct mitosomal localization for all 4 HA-tagged variants had been previously confirmed by IFA (Fig 4 and S1C–S1F Fig).

#### Expansion of the mitosomal membrane interactome network beyond and within the outer mitosomal membrane with MOMTiP-3, MOMTiP-4 and MOMTiP-7

**Fig 6:**
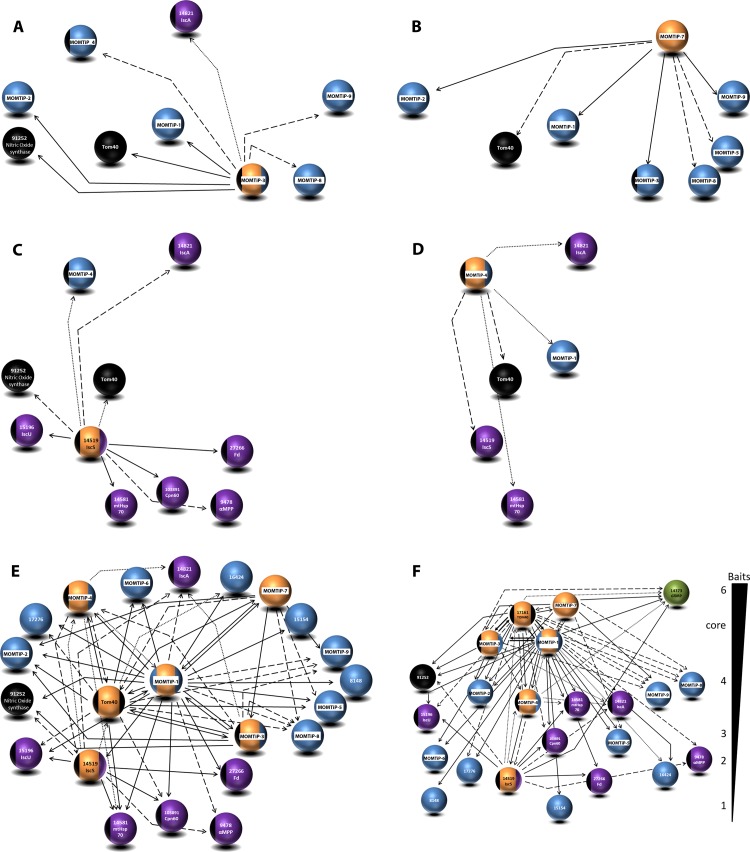
Expansion of the core interactome beyond the outer mitosomal membrane with co-IPs of HA-tagged MOMTiP-7, MOMTiP-4, MOMTiP-3 and GlIscS. Epitope (HA)-tagged (A) MOMTiP-3, (B) MOMTiP-7, (C) GlIscS and (D) MOMTiP-4 –derived interactomes. (E, F) Alternative depictions of the cumulative interactome of proteins localizing to mitosomes generated with 6 bait proteins. Note the tight association of GlTom40 with MOMTiP-1 and MOMTiP-3. (F) A socio-affinity depiction of protein-protein interactions derived from all co-IP experiments. Proteins are grouped according to how many baits they were significantly associated with. GlDRP (green sphere) is pulled down with all 6 bait proteins used in the co-IP assay at different stringency parameters. Bait proteins (orange spheres), matrix proteins (purple), previously identified and localized proteins (black), and localized hypothetical proteins (blue). The stringency parameters used for detection (high, medium, and relaxed) are represented by bold, dashed, and dotted arrows, respectively.

**MOMTiP-3**, also known as MOMP35 [49], is predicted to contain 2 TMDs and was analyzed because of its potential role as a component of the import complex [49]. Co-IP using HA-tagged MOMTiP-3 as bait yielded 93 bait-specific candidate interactors. Both *Gl*Tom40 and *G*. *lamblia* oxidoreductase 1 (GiOR1) were detected in this dataset, in addition to several previously identified hypothetical mitosomal proteins (e.g. *Gl50803_29147*, *Gl50803_10971* and *Gl50803_7188*) (Fig 6A), suggesting that MOMTiP-3 is a significant interacting partner of *Gl*Tom40 and MOMTiP-1.

Consistent with its prediction as a SNARE protein, **MOMTiP-7** presents dual localization, (mitosome and ER; Fig 4H and S1C Fig) and was identified as a strong interactor in both the *Gl*Tom40 and MOMTiP-1 co-IP datasets. We postulated that MOMTiP-7 may have a role in inter-organelle communication between mitosomes and the ER, possibly in protein/lipid transport [68,69]. We reasoned that identifying interaction partners could shed light on the nature of physical contacts between mitosomes and other membrane-bounded compartments. Analysis of co-IP using HA-tagged MOMTiP-7 as bait yielded 157 bait-specific proteins. Interestingly, the only 2 proteins in the dataset with high spectral counts were the bait itself and MOMTiP-3. Several non-annotated proteins, e.g. MOMTiP-2, MOMTiP-1, MOMTiP-9, *Gl*Tom40 as well as Type III DnaJ protein *Gl50803_9751* were detected with relaxed stringency parameters.

**MOMTiP-4** is a predicted soluble *Giardia*-specific protein of unknown function with a mitochondrial targeting signal. In line with this, HA-tagged MOMTiP-4 localized exclusively to mitosomes (Fig 4E and S1E Fig). MS data analysis performed with high stringency parameters of a MOMTiP-4 co-IP experiment (95_2_95; FDR 0%) yielded only 12 bait-specific hits, none of them known mitosome proteins. The bait protein itself was by far the most significant hit in the dataset. Analysis with more relaxed stringency parameters (90_1_90; FDR 6.2%) yielded 47 bait-specific identifications which included matrix proteins *Gl*IscS, *Gl*IscA, *Gl*Hsp70, and also *Gl*Tom40. The MOMTiP-4 co-IP dataset suggests that despite its clear-cut localization and considerable expression levels judged by the signal obtained in Fig 4E, this mitosomal protein has a limited interactome enriched mostly in matrix proteins. MOMTiP-1was identified only at very low stringency (20_1_20; FDR 51%) in this dataset, suggesting that MOMTiP-4 and MOMTiP-1 are not direct interactors but could be connected via bridging proteins. All identified MOMTiP-4-interacting proteins are depicted in Fig 6D.

#### Expansion of the the GlTom40 interactome towards the matrix with GlIscS

**GlIscS** is a mitosomal matrix protein and the central component of the Fe-S assembly machinery [70]. All mitosomal matrix proteins including GlIscS are translated in the cytoplasm and reach their final destination after unfolding and translocation across the mitosome double membrane. Thus, this trafficking route (cytoplasm–translocon–matrix) should be reflected in the protein-protein interactions of a co-IP dataset with GlIscS-HA as bait. Co-IP of HA-tagged GlIscS yielded 177 bait-specific protein hits. Among these, we identified all 5 known matrix proteins namely, NifU-like protein, HSP70, [2Fe-2S] ferredoxin, Cpn60, and GiOR1. GlTom40 as the sentinel protein for the outer membrane translocon was detected with relaxed stringency (50_1_50, FDR of 30%). Seventy out of 177 hits were enriched in the GlIscS-specific dataset, with ≥ 5 peptide counts. Eighteen of those (25%) belong to the Protein 21.1 family. The biological function of this protein family in G. lamblia [71] and the significance of its association to GlIscS is unknown.

In summary, we have generated an extensive mitosome-centered protein interaction network (Fig 6E) from 6 independent co-IP assays using epitope-tagged *Gl*Tom40 and 5 interaction partners (MOMTiP-1, MOMTiP-3, MOMTiP-7, *Gl*IscS and MOMTiP-4) as baits based on i) spectral counts with high stringency parameters in the *Gl*Tom40 co-IP dataset and ii) confirmed localization of epitope-tagged variants to mitosomes by IFA. All 24 localized mitosome proteins (previously known and newly identified hypotheticals) were parsed according to molecular function and biological process (S3 Fig) using Blast2go (https://www.blast2go.com/). Metal ion, Fe-S, ATP, and protein binding were the major molecular functions associated with these proteins. Interestingly, other biological processes involving response to lipid and transmembrane transport were also identified with significant p-values. An additional 93 candidates annotated as hypothetical proteins (from all the 6 co-IP assays) were analyzed using Blast2go (S4 Fig). Binding and catalytic activities were the 2 major GO terms associated to this group. Interestingly, *Gl*DRP was strongly overrepresented in 3 high-stringency co-IP datasets where mitosome membrane proteins were specifically used as bait. Moreover, with relaxed stringency parameters *Gl*DRP was detected in all 6 co-IP datasets (Fig 6F), indicating that *Gl*DRP is associated to mitosomes. These data are clearly in line with our previous observations concerning the perturbation of mitosome morphogenesis by mutant *Gl*DRP (Fig 2D–2F). In Table 2, we have combined our data with data reported in [33,49] for a state-of-the-art overview of the main confirmed interactions within the mitosome-centered protein interactome.

**Table 2:** Detected and validated mitosomal proteins in this report and in [33,49,72].

Assigned annotation on www.giardiaDB.org and/or ORF number	Detection of annotated and novel mitosomal proteins in this work, co-IPed with epitope-tagged						Detection of annotated and novel mitosomal proteins in Martincova et.al 2015 using BirA fusions to					Detection of annotated and novel mitosomal proteins in		
	Tom40	MOMTiP-1 (GL50803_ 29147)	MOMTiP-3 (GL50803_ 14939)	MOMTiP-5 (GL50803_ 5785)	MOMTiP-4 (GL50803_9296)	*Gl*IscS	Pam18	Tim44	Hsp70	Tom40	Gl14939 (GiMOMP35)	Jedelsky et. al, 2011	Martincova et. al, 2105	This work
**Tom40 (GL50803_17161)**														
**GL50803_14939 (GiMOMP35)**														
**Pam 18 (GL50803_300001)**														
**Pam 16 (GL50803_19230)**														
**Tim44 (GL50803_14845)**														
**mtHSP70 (GL50803_14581)**														
**Mge1 (GL50803_1376)**														
**Isc S (GL50803_14519)**														
**IscU (GL50803_15196)**														
**IscA (GL50803_14821)**														
**DnaJ (GL50803_17030)**														
**DnaJ (GL50803_9751)**														
**αMPP (GL50803_9478)**														
**GiOR-1 (GL50803_91252)**														
**Glutaredoxin5 (GL50803_2013)**														
**Ferredoxin (GL50803_27266)**														
**Cpn60 (GL50803_103891)**														
**Cpn10 (GL50803_29500)**														
**GrpE (GL50803_1376)**														
**GL50803_29147**														
**GL50803_9296**														
**GL50803_10971**														
**GL50803_5785**														
**GL50803_7188**														
**GL50803_9503**														
**GL50803_21943**														
**GL50803_22587**														
**GL50803_17276**														
**GL50803_16424**														
**GL50803_8148**														
**GL50803_15154**														

The presence (in light green) of any given protein was based on stringency thresholds specific to each report. From darker to increasingly lighter shades of grey, proteins are putatively grouped in outer membrane, inner membrane, matrix and novel mitosomal components.

### A pharmacologically-induced mitosome matrix-targeted DHFR complex inhibits processing of an imported endogenous reporter in mitosomes

**Fig 7:**
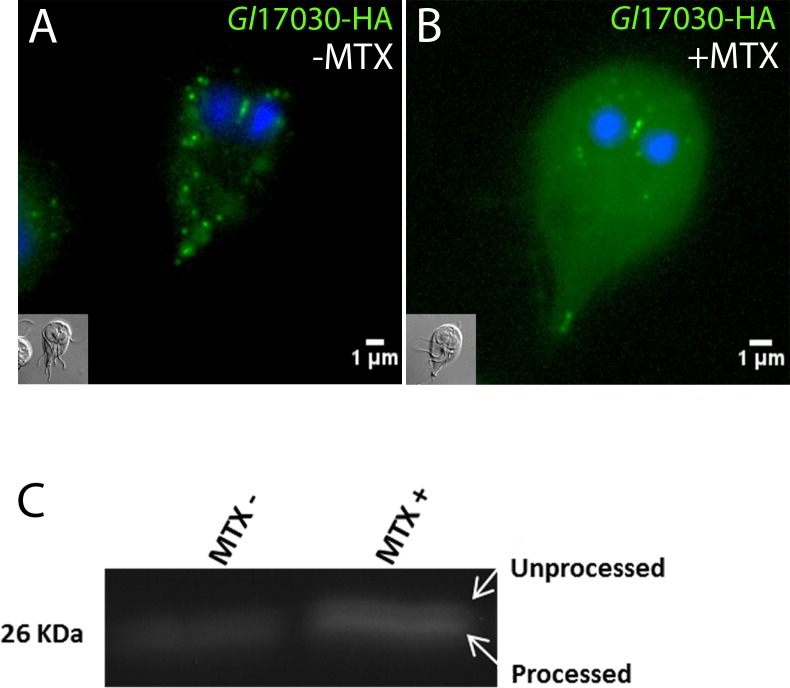
MTX treatment of cells expressing mitosome-targeted DHFR affects processing of a mitosomal matrix reporter. Subcellular distribution of a matrix-targeted reporter (*Gl*17030-HA) without MTX (A) or after addition of 1μM MTX for 24 h (B) in transgenic cells expressing mitosome-targeted DHFR. Note the accumulation of HA-signal in the cytoplasm. Nuclear DNA is stained with DAPI (blue). Insets: DIC images. (C) Immunoblot analysis detects accumulation of unprocessed *Gl*17030-HA in the presence of MTX.

Evidence from extensive primary and reverse co-IP data combined with IFA analysis led us to postulate that *Gl*Tom40, MOMTiP-1 and MOMTiP-3 exist in an outer membrane core complex, likely involved in protein import. We probed the functional conservation of mitosomal import across the *Gl*Tom40 translocon with respect to the corresponding process in *bona fide* mitochondria by adapting the DHFR-folate analogue system [49,73] to *G*. *lamblia*. Pre-sequence directed DHFR is a classical substrate used in protein translocation studies due to its ability to fold irreversibly upon binding a folate analog, e.g. MTX. Complexed with MTX, DHFR becomes unsuitable as a substrate for import and blocks translocons, which results in a general blockage of organelle protein import [73]. Transfection of MTSfdΔ_int_-DHFR into a *Gl*17030-HA background, i.e. a transgenic line expressing an HA-tagged MTS-directed mitosomal reporter, allowed testing of the general effects of MTX-induced import block. We reasoned that the presence of MTX in MTSfdΔint-DHFR expressing cells could lead to an import block due to jamming of the translocase. Localization of the reporter by IFA showed an increased cytosolic *Gl*17030-HA signal after addition of 1 μM MTX (Fig 7B) compared to parasites exposed to the solvent alone (Fig 7A). This suggested accumulation of the reporter in the cytosol in cells exposed to MTX as a result of a generalized import block. To test this we measured the ratio of the slightly larger *Gl*17030-HA reporter precursor protein and the imported and therefore processed form without the MTS by SDS-PAGE and Western blot using anti-HA antibodies. Consistent with the IFA data, unprocessed *Gl*17030-HA was strongly increased in the drug treated sample, whilst only the processed form was present in untreated controls (Fig 7C). Taken together the data support functional conservation of the highly diverged protein import machinery in *G*. *lamblia* mitosomes.

## Discussion

**Fig 8:**
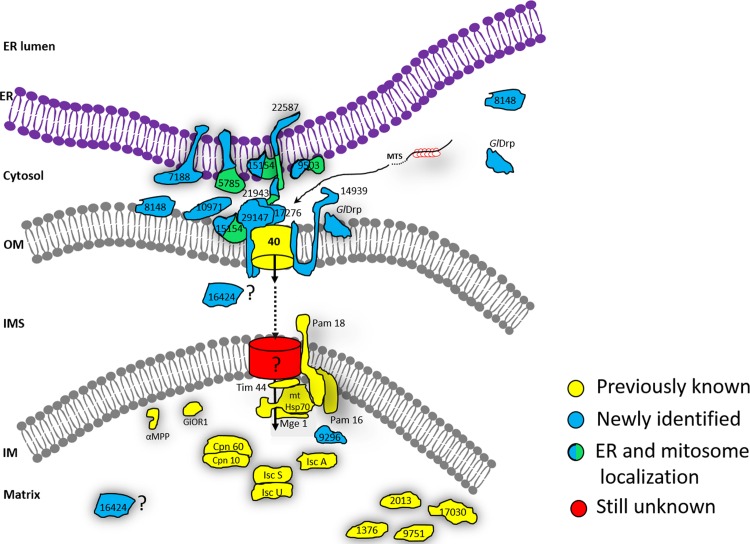
An integrated model for mitosome interactome networks. Schematic representation of all proteins identified via protein-protein interaction data through serial coIP assays using 6 different mitosomal bait proteins are shown in a model. Previously identified/ known mitosomal proteins are depicted in yellow. Newly identified mitosome localized hypothetical proteins are shown in blue. Proteins with dual localization (mitosomes and ER) are shown in blue/green. As yet un-identified pore (translocase) in the inner membrane is shown in red. Positioning of these identified proteins on the model is based on *in silico* data (presence/ absence of (1) mitochondrial targeting sequence, (2) transmembrane domain), localization data and socio- affinity interaction of these proteins with their respective and other bait proteins.

*G*. *lamblia* mitosomes remain the smallest known and least characterized MROs. Identification of protein components using shotgun proteomic analysis of enriched mitosome preparations has proven challenging primarily due to difficulties in isolating sufficient amounts of contaminant-free organelles [33,48]. Extensive sequence divergence prevents identification of organelle proteins *via* homology-based searches; a case in point is *Gl*TOM40 whose sequence degeneration is so extensive that the identification of orthologues in *Giardia*, *Entamoeba* or *Spironucleus* remains tentative despite the constraints imposed by the beta barrel structure of these mitochondrial porins [44]. The function of candidate factors identified by other means and localized to organelle membranes usually cannot be deduced based on existing structural information from well-characterized mitochondrial homologs. A notable exception to this is a recently identified highly-diverged but structurally-conserved *Gl*Tim44 homologue [49]. Taken together, these challenges have frustrated attempts at systematizing intra- and inter- organelle mitosome-centered interactions, thereby limiting analysis to isolated complexes [49,50]. For example attempts at analyzing isolated GlTom40-containing protein complexes (presumably enriched mitosomal outer membrane translocons) using blue-native PAGE [50] detected no mitosomal proteins aside from GlTom40 and an unidentified 32kDa protein which could not be mapped to any known Giardia sequence. These data demonstrate how challenging it is to define novel GlTom40-interacting partners, probably due to the translocon complex being embedded in the outer membrane of the organelle. Here, we used epitope-tagged *Gl*TOM40 [23,33] as first bait, and implemented an iterative co-immunoprecipitation approach to expand the mitosomal membrane interactome network beyond the few known components. With only 5 more bait proteins, this strategy allowed for building of a core membrane interactome and a complex interactome network extending inwards to the organelle matrix as well as outwards to components of the ER membrane, the axoneme cytoskeleton and the cytoplasm. The rationale is that with sufficient numbers of targeted reverse co-IP experiments using validated organelle proteins as baits, a comprehensive proteome interactome could be built, thereby achieving a systems-biological view of the giardial mitosome proteome. From a technical point of view, building an interactome network using a forward- and reverse co-IP approach allows isolation of “true” mutual interactions by validation in two completely independent co-IP experiments. Specifically, the 108 MS hits detected in the Tom40 co-IP dataset which include numerous non-specific interactions can be filtered with data from reverse co-IP assays to reveal actual protein-protein interactions (depicted in Fig 6E) that can be unambiguously distinguished from false-positive hits. Combined with imaging data and predicted topology this provides a robust platform to construct an integrated working model of all mitosome-associated protein interactome networks known to date (Fig 8). Blast2Go *in silico* enrichment analyses suggest that mitosomes may have a role beyond Fe-S protein maturation (S3 and S4 Figs) Only recently the major function of *E*. *histolytica* mitosomes was shown to be sulfate activation, and not Fe-S protein maturation as previously thought [44]. Although genes involved in this pathway are missing in other MRO-containing organisms such as *G*. *lamblia*, *T*. *vaginalis*, and *C*. *parvum*, the *Entamoeba* example points to a wider range of functions ascribable to mitosomes. This may even include general functions in stage-differentiation as recently shown in *E*. *histolytica* whose mitosomes are essential for the encystation process [74].

### *Gl*Tom40 and interaction partners MOMTiP-1 and MOMTiP-3: a minimized mitosome protein import apparatus?

Following its identification as a prominent *Gl*Tom40 interaction partner, the single pass membrane protein MOMTiP-1 was the first bait protein selected for reverse co-IP to expand the *Gl*Tom40 interactome. MOMTiP-1 as bait pulled down *Gl*Tom40 with the most abundant peptide counts. GFP-tagging and detection of MOMTiP-1::GFP on mitosomes suggests a membrane topology in line with characterized mitochondrial receptor proteins such as Tom20 [75]. Further support for MOMTiP-1’s membrane topology may derive from a definition of membrane orientation using alternative methods such as in situ proximity ligation or protease protection assays. The latter approach has proven useful in the determination of membrane topology for other mitosomal candidate proteins in Giardia [49]. The identification of MOMTiP-1 provides an exciting lead; however, a detailed functional characterization of this protein is required to provide independent evidence for the exact nature of this interaction and to test the hypothesis that MOMTiP-1 is a component of the *Gl*Tom40 complex with a receptor function. So far 20 proteins have been validated by localization to mitosomes, allowing for a significant expansion of the *Gl*Tom40/MOMTiP-1 interactome. This protein’s predicted topology combined with its exclusive mitosomal localization and the size and composition of its interactome, supports MOMTiP-1 as a *Gl*Tom40 accessory protein with a potential receptor function for protein import. To test this hypothesis, we engineered a truncated HA-tagged version of MOMTiP-1 consisting only of the predicted C-terminal domain (residues 31–133; C-MOMTiP-1). Ectopic expression of C-MOMTiP-1 showed a distinct cytosolic localization by IFA (S5A Fig). Native co-IP of C-MOMTiP-1 and analysis of the bait-specific dataset with medium stringency parameters (95_1_95) identified only 2 mitosomal proteins (Gl50803_16424 and MOMTiP-8) (S5B Fig). These data show that the soluble cytoplasmic MOMTiP-1 variant does not recapitulate the interaction properties of the full-length membrane-anchored protein, suggesting that capture of imported matrix proteins may require incorporation of the putative receptor domain into a fully-assembled TOM complex, complete with ancillary factors.

MOMTiP-3 was exclusively identified in the *Gl*Tom40 and the MOMTiP-1 co-IP datasets, suggesting that these 3 proteins may function in a tightly-knit complex, likely involved in protein import across the outer mitosomal membrane. TMHMM predicts two TMDs at MOMTiP-3’s N-terminus, followed by a large C-terminal domain. Powerful HMMER-based searches across several eukaryotic lineages, including the closely related diplomonad *Spironucleus salmonicida* [76], yielded no orthologues for MOMTiP-3, neither was there any predicted functional information available. Nevertheless, analysis of protease protection assays for this protein showed that MOMTiP-3 localizes at the outer mitosome membrane with its C- terminus in the cytosol [49]. These data, in combination with data on MOMTiP-1 predicted topology and interactomes developed herein, support a model for *Gl*Tom40, MOMTiP-1, and MOMTiP-3 for a minimized mitosomal import apparatus whose core import machinery is composed of only these 3 proteins. The dramatic perturbation of mitosomal homeostasis observed when either MOMTiP-1 (this work) or MOMTiP-3 [49] were constitutively overexpressed supports the hypothesis for their belonging to the same complex. Protein translocation across the outer mitosomal membrane through this highly reduced import apparatus would be conserved in its mechanism, given that MTX-induced complexing of mitosome-targeted DHFR caused accumulation of unprocessed i.e. untranslocated mitosome reporters (Fig 7C, [49]). Incidentally, these data also confirm that mitosome membrane translocation requires pre-proteins to remain in an unfolded state [49].

### Mitosome-ER contact sites

Co-IP data combined with imaging of tagged variants identified 6 proteins with dual localization at mitosomes and ER (Fig 8). Contact between these organelles would serve at least two major functions, i.e. replication of mitosomes and transport associated to lipid biosynthesis. Thus far, we have identified five mitosome proteins with dual localization potentially involved in inter-organelle communication (Fig 4F). One of them is a transmembrane Qb-SNARE 4 (MOMTiP-7) [65] identified in *Gl*Tom40 and MOMTiP-1 co-IP datasets.

For their biogenesis, mitochondria and MROs rely on lipid transfer from the ER, the central site for phospholipid synthesis in the cell [77,78]. SNAREs are best known for mediating membrane fusion in vesicular transport [79] whereas in the context of mitochondria and the ER, they function as components of so called ER-mitochondria encounter structures (ERMES). In addition to being associated to mitochondrial protein import [80,81], ERMES fulfills an essential function in inter-organelle lipid transport [80]. Phosphatidylserine is shuttled from the ER to mitochondria through the ERMES complex where it is converted to phosphatidylethanolamine (PE) by a decarboxylation reaction that generates most if not all PE in mitochondria [80,82]. Unlike in the hydrogenosome-containing *T*. *vaginalis* [83], ERMES homologs have not been identified in *G*. *lamblia*, possibly due to extensive sequence divergence. Thus, whether this function is preserved in *Giardia* mitosomes is not known however, organelle biogenesis would necessarily depend on ER-derived lipids which are transported to mitosomes either by carrier proteins or via membrane contact sites. The latter requires tethering complexes to facilitate phospholipid exchange between the two organelles. Given that MOMTiP-7 is predicted to be a SNARE, we explored the idea that this protein is part of a larger complex mediating ER-mitosome interaction. Co-IP of MOMTiP-7 specifically detected, in addition to outer membrane proteins such as *Gl*Tom40, MOMTiP-1 and MOMTiP-3, 3 hypothetical proteins, two of which, MOMTiP-8 and MOMTiP-5 (both predicted soluble proteins), localized both to the ER and to mitosomes. In addition, a domain in MOMTiP-8 has similarity to a yeast “Maintenance of mitochondrial morphology” protein 1 (Mmm1) of the ERMES complex. Moreover, HHpred analysis revealed a link between MOMTiP-5 and a beta barrel lipid binding protein MLN64 (e-value 0.0006) in *H*. *sapiens* which facilitates cholesterol transport to mitochondria [84]. These preliminary data support the existence of an outer mitosomal membrane-associated complex in *G*. *lamblia* mitosomes possibly involved in generating ER—mitosome membrane contact sites (Fig 8).

### Mitosome dynamics and a novel role for MOMTiP-1 and *Gl*DRP in mitosome homeostasis

We had previously shown that replication and inheritance of the CMC is coordinated in a cell cycle-dependent manner, whereas PMs divided stochastically [23]. The lack of a system to track organelles in living trophozoites precluded addressing the question directly whether mitosomes were motile and constituted a dynamic network of organelles with measurable exchange. Development of two GFP-tagged reporters GFP-*Gl*Tom40 and MOMTiP-1-GFP (this study) allowed for time-lapse experiments to follow individual organelles in a cell. However, we found no evidence for motility of organelles, neither in the CMC nor in PMs, even after prolonged observation (1.5 h). This is consistent with the lack of motor proteins such as kinesins and dyneins in any of the mitosomal protein interactomes we generated. Moreover, FRAP experiments revealed no exchange of GFP-tagged membrane proteins between organelles during the period of observation (Fig 7F and 7G), which further corroborated the relative isolation of mitosomes within the cytosol. The lack of mitosomal motility and contact complicates investigation of their replication and morphogenesis. The two most plausible scenarios for this are currently the following: i) PMs are released from the CMC, which continuously produces new organelles by elongation and fission to maintain a constant number of organelles in a cell-cycle independent manner; ii) PMs and the CMC organelles replicate independently in a cell-cycle independent and -dependent manner, respectively [23]. Although time-lapse microscopy experiments did not provide evidence in support of either scenario, conditional expression of a dominant-negative, constitutively active *Gl*DRP-K43E revealed a distinct morphogenesis phenotype (see also below) indicative of an organelle replication defect. As one of the key players in the regulation of mitochondrial fission, DRPs are mechano-enzymes conserved from yeast to vertebrates [85,86,87,88]. *G*. *lamblia* harbors a single dynamin homologue *Gl*DRP shown to play a major role in this parasite’s endocytic pathway and stage conversion [56,89,90]. Transgenic parasites expressing the *Gl*DRP-K43E variant exhibited larger and fewer mitosomes, compared to cells expressing the wild type *Gl*DRP variant(Fig 6). This is in line with the dominant-negative effect on mitochondrial fission elicited by the corresponding mutation in DRPs in other organisms. To our knowledge, this is the first report on the involvement of *Gl*DRP in mitosome homeostasis, supporting the (at least partial) functional conservation of mitochondrial and MRO fission [91,92,93,94]. The notion that *G*. *lamblia* mitosome fission is functionally conserved is further substantiated by the identification of *MOMTiP-6* which presents dual localization to mitosomes and the ER. HMMER-based predictions relate *MOMTiP-6* to human mitochondrial fission protein (Fis1, e-value 6.3E-05) which participates in the recruitment of dynamin-related protein 1 (Drp1) to the mitochondrial surface for organelle fission [95,96]. The distinctive “string” mitosome phenotype in cells expressing MOMTiP-1-GFP clearly demonstrated that mitosomes can assume an elongated, tubular morphology, which is a prerequisite for organelle division and replication. The implication is that *G*. *lamblia* mitosomes retain at least the machinery for fission in which the mechano-enzyme *Gl*DRP and outer mitosomal membrane elements such as MOMTiP-1 and 3 [49] play central roles.

## Conclusion

We used an iterative approach based on co-IP experiments to generate a *Gl*Tom40-centered interactome network. Ultimately this strategy should allow building a combined proteome, which delineates the full complement of organelle proteins, peripherally associated factors, as well as interfaces with the ER and the cytoskeleton. Although this strategy requires numerous rounds of sequential co-IP and validation, it is highly informative because it produces interaction data in addition to identifying novel organelle proteins. Combined with testing of epitope-tagged variants of candidate proteins for organelle localization as a straightforward validation criterion, serial co-IPs allow for unambiguous definition of the organelle-specific proteome, as well as interfaces with other cellular structures. This strategy also led to the discovery of MOMTiP-1, a strong *Gl*Tom40 interaction partner which plays a role in mitosomal morphogenesis. Together with *Gl*DRP (this work) and MOMTiP-3 (MOMP35; [49]), these are the only proteins so far known to affect mitosomal homeostasis in *G*. *lamblia*.

## Supporting Information

S1 Table Oligonucleotides used in this study. (XLSX)

S2 Table MOMTiP-1 to 13 spectral counts with high stringency parameters, available annotation and experimentally-verified subcellular distribution. (XLSX)

S1 Fig Co-labelling of HA-tagged co-IP baits with the mitosomal marker *Gl*IscU. Immunofluorescence co-labelling and wide-field microscopy analysis of transgenic *G*. *lamblia* lines expressing (A) *Gl*Tom40, (B) MOMTiP-1, (C) MOMTiP-7, (D) *Gl*IscS, (E) MOMTiP-4 and (F) MOMTiP-3, all used as HA-tagged baits in co-IP experiments (upper row, in green), in combination with the endogenous mitosomal marker *Gl*IscU (middle row, in red). Nuclei were stained with DAPI (lower row, in blue). The central mitosome complex was clearly labelled by both fluorophores in all lines. Scale bar: 1μm.
(TIF)

S2 Fig Titration assay to determine optimum crosslinker concentration for co-IP experiments. With increasing concentrations of formaldehyde (0–4.5%), immuno-detection (Western blot) of the Tom40-HA reporter shows a shift from the monomeric form to higher molecular weight complexes,. Molecular size (kDa) marker (M) bands are indicated on the left axis. A concentration of 2.25% formaldehyde (arrowhead) was later adopted for all subsequent forward and reverse co-IPs.
(TIF)

S3 Fig Blast2Go analysis for 26 mitosome localized proteins. (A) Data distribution pie chart for protein hits with either Blast2Go annotation (“B2G annotated”), or associated to gene ontology (GO) terms (“with GO mapping”) or to Blast annotation data (“with Blast hits”). (B) The top 20 GO terms distributed in the three root categories for “biological process” (BP), “molecular function” (MF) and “cellular component” (CC). (C) A direct count of GO terms associated to MF. (D) A direct count of GO terms associated to BP.
(TIF)

S4 Fig Blast2Go analysis for 93 hypothetical mitosomal proteins from 6 co-IP assays. (A) Data distribution pie chart for protein hits with either Blast2Go annotation (“B2G annotated”), or associated to gene ontology (GO) terms (“with GO mapping”) or to Blast annotation data (“with Blast hits”). (B) The top 20 GO terms distributed in the three root categories for “biological process” (BP), “molecular function” (MF) and “cellular component” (CC). (C) A direct count of GO terms associated to MF. (D) A direct count of GO terms associated to BP.
(TIF)

S5 Fig IFA and native co-IP analysis of the predicted C-terminal domain of MOMTiP-1. (A) IFA and wide-field microscopy analysis of transgenic Giardia cells expressing C-MOMTiP-1 (in green), a truncated HA-tagged version of MOMTiP-1 consisting only of the predicted C-terminal domain (residues 31–133). C-MOMTiP-1 accumulates primarily in the cytosol. Nuclei are labelled with DAPI (in blue). Inset: DIC image. Scale bar: 1μm. (B) Venn diagram depicting the overlap of datasets derived from native co-IP of C-MOMTiP-1 and control WB cells.
(TIF)
