# Supplementary figures and images for: An Interactome-Centered Protein Discovery Approach Reveals Novel Components Involved in Mitosome Function and Homeostasis in Giardia lamblia
Source: PLoS Pathog. 2016 Dec 7;12(12):e1006036. doi: 10.1371/journal.ppat.1006036 (PMC5142787; doi:10.1371/journal.ppat.1006036)

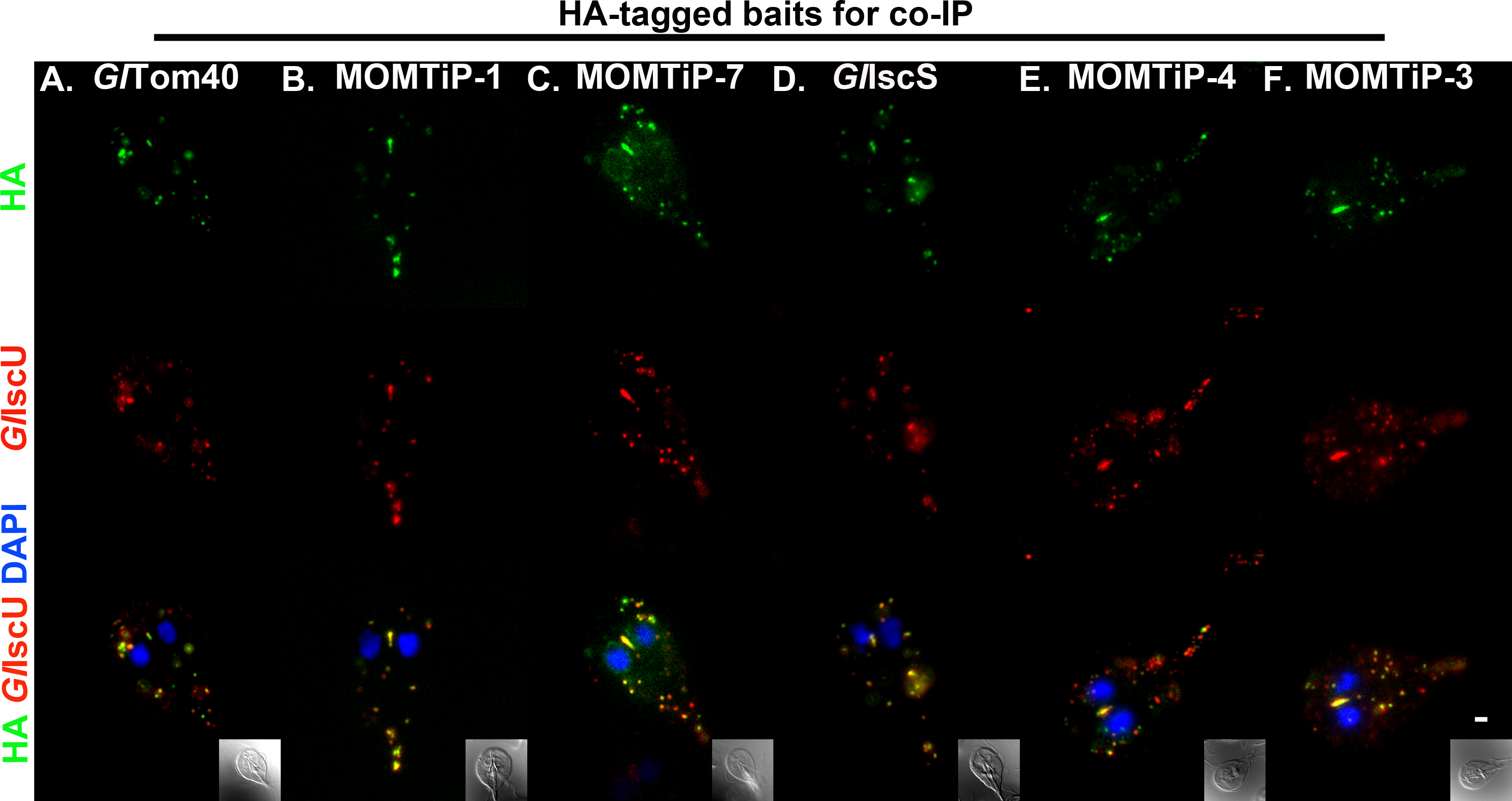

Supplement: S1 Fig — Immunofluorescence co-labelling and wide-field microscopy analysis of transgenic G. lamblia lines expressing (A) GlTom40, (B) MOMTiP-1, (C) MOMTiP-7, (D) GlIscS, (E) MOMTiP-4 and (F) MOMTiP-3, all used as HA-tagged baits in co-IP experiments (upper row, in green), in combination with the endogenous mitosomal marker GlIscU (middle row, in red). Nuclei were stained with DAPI (lower row, in blue). The central mitosome complex was clearly labelled by both fluorophores in all lines. Scale bar: 1μm. (TIF) [file ppat.1006036.s003.tif]

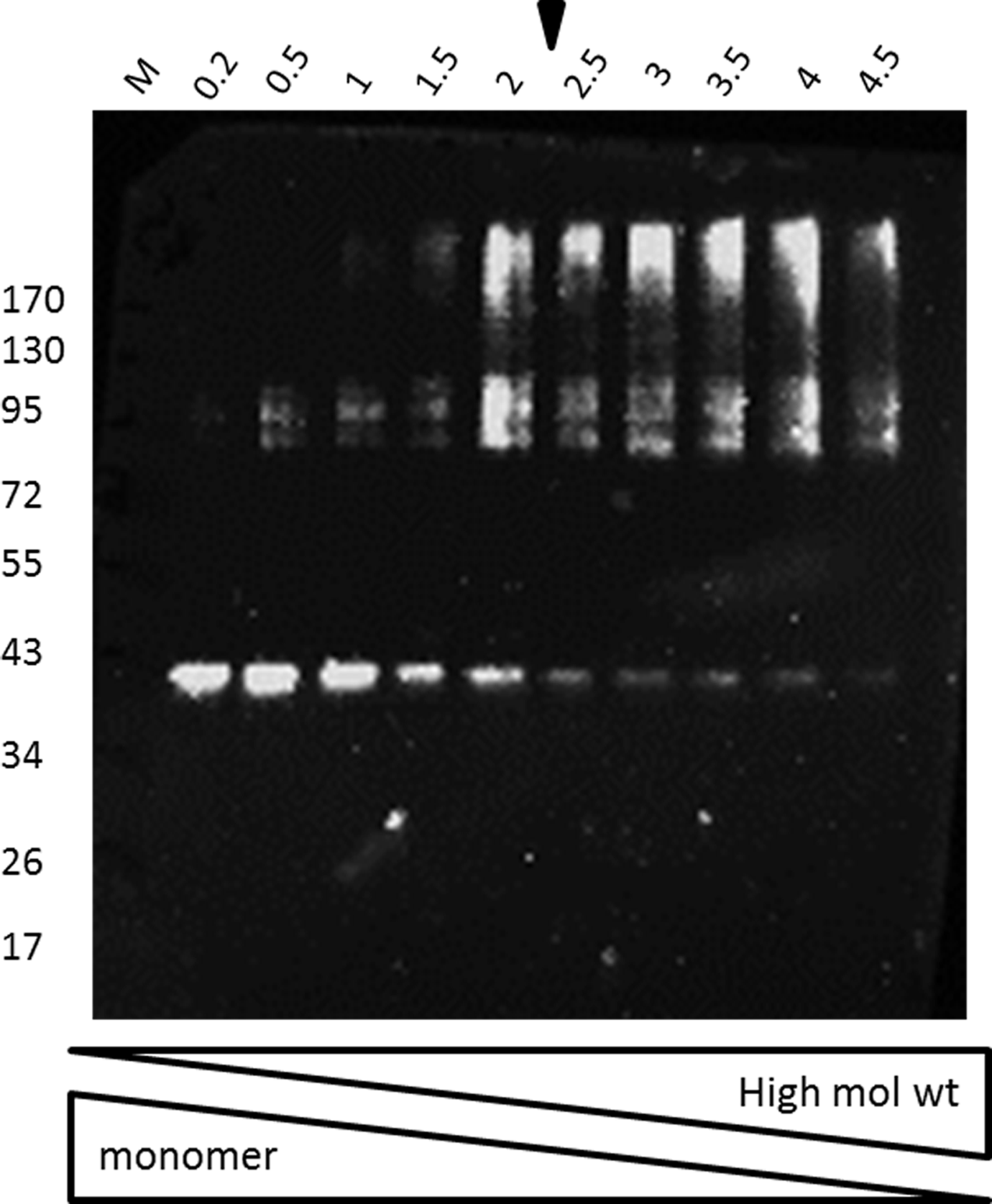

Supplement: S2 Fig — With increasing concentrations of formaldehyde (0–4.5%), immuno-detection (Western blot) of the Tom40-HA reporter shows a shift from the monomeric form to higher molecular weight complexes,. Molecular size (kDa) marker (M) bands are indicated on the left axis. A concentration of 2.25% formaldehyde (arrowhead) was later adopted for all subsequent forward and reverse co-IPs. (TIF) [file ppat.1006036.s004.tif]

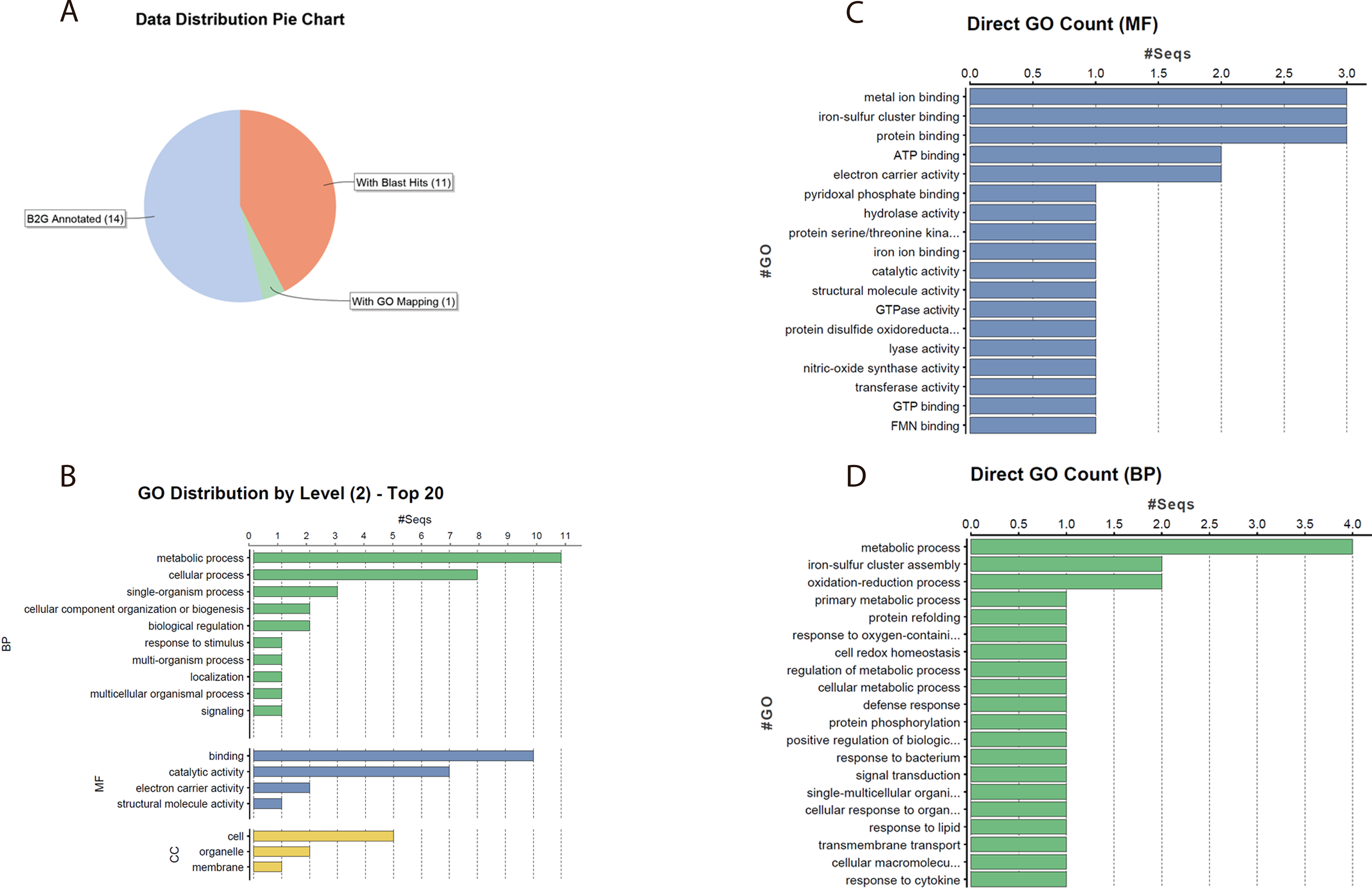

Supplement: S3 Fig — (A) Data distribution pie chart for protein hits with either Blast2Go annotation (“B2G annotated”), or associated to gene ontology (GO) terms (“with GO mapping”) or to Blast annotation data (“with Blast hits”). (B) The top 20 GO terms distributed in the three root categories for “biological process” (BP), “molecular function” (MF) and “cellular component” (CC). (C) A direct count of GO terms associated to MF. (D) A direct count of GO terms associated to BP. (TIF) [file ppat.1006036.s005.tif]

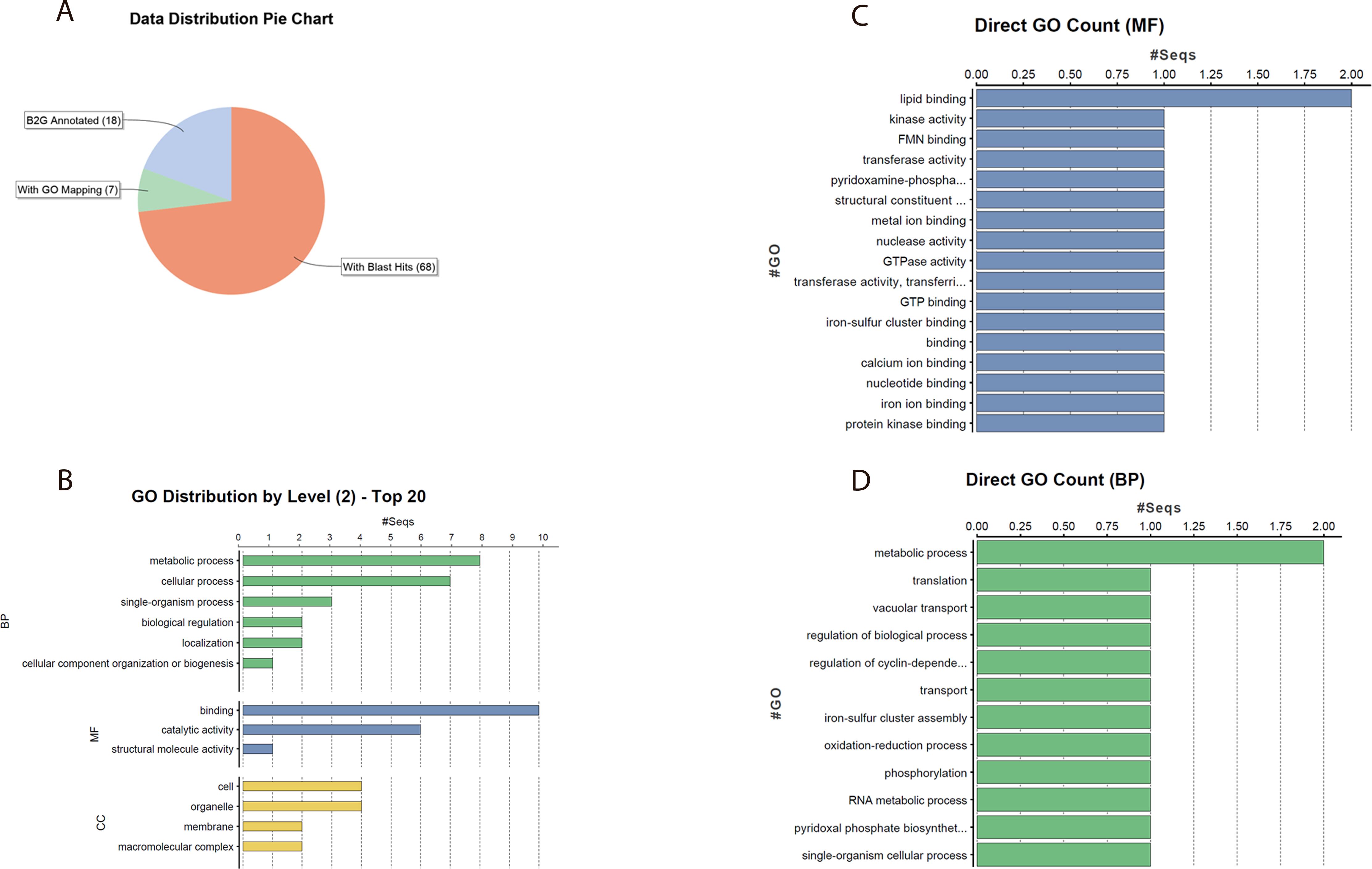

Supplement: S4 Fig — (A) Data distribution pie chart for protein hits with either Blast2Go annotation (“B2G annotated”), or associated to gene ontology (GO) terms (“with GO mapping”) or to Blast annotation data (“with Blast hits”). (B) The top 20 GO terms distributed in the three root categories for “biological process” (BP), “molecular function” (MF) and “cellular component” (CC). (C) A direct count of GO terms associated to MF. (D) A direct count of GO terms associated to BP. (TIF) [file ppat.1006036.s006.tif]

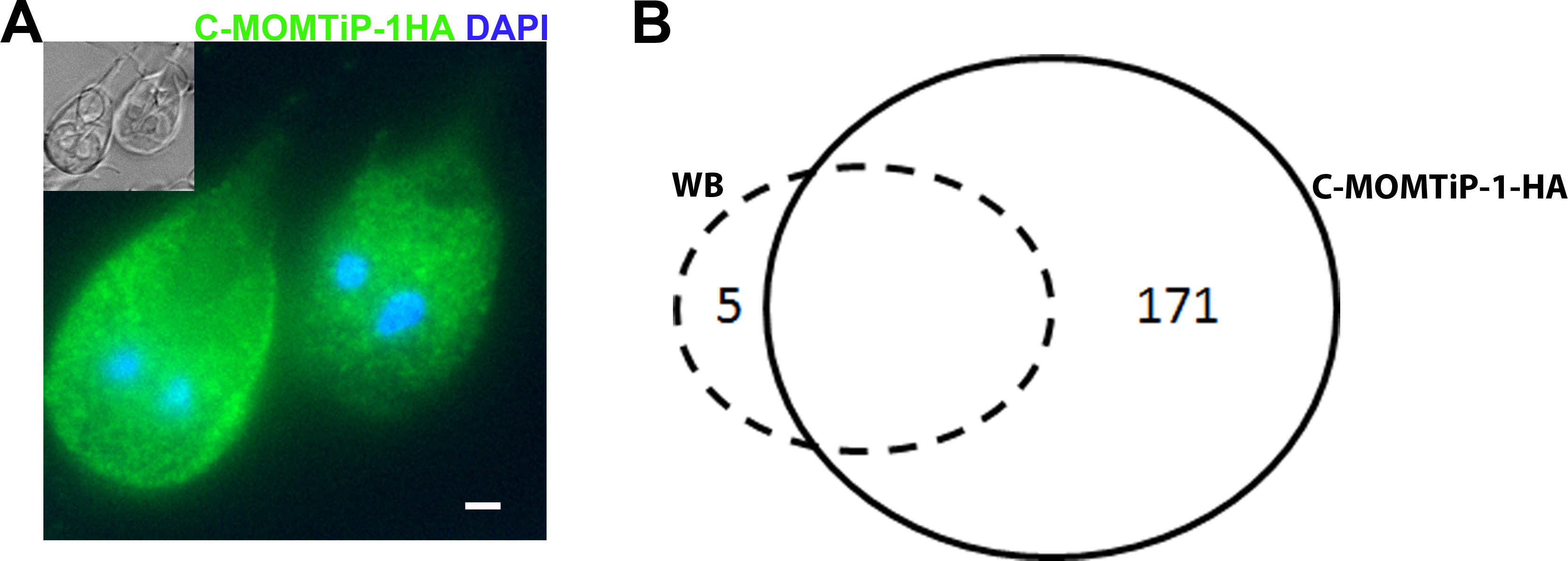

Supplement: S5 Fig — (A) IFA and wide-field microscopy analysis of transgenic Giardia cells expressing C-MOMTiP-1 (in green), a truncated HA-tagged version of MOMTiP-1 consisting only of the predicted C-terminal domain (residues 31–133). C-MOMTiP-1 accumulates primarily in the cytosol. Nuclei are labelled with DAPI (in blue). Inset: DIC image. Scale bar: 1μm. (B) Venn diagram depicting the overlap of datasets derived from native co-IP of C-MOMTiP-1 and control WB cells. (TIF) [file ppat.1006036.s007.tif]
